# Counting on β-Diversity to Safeguard the Resilience of Estuaries

**DOI:** 10.1371/journal.pone.0065575

**Published:** 2013-06-05

**Authors:** Silvia de Juan, Simon F. Thrush, Judi E. Hewitt

**Affiliations:** National Institute of Water and Atmospheric Research, Hamilton, New Zealand; University of Florida, United States of America

## Abstract

Coastal ecosystems are often stressed by non-point source and cumulative effects that can lead to local-scale community homogenisation and a concomitant loss of large-scale ecological connectivity. Here we investigate the use of β-diversity as a measure of both community heterogeneity and ecological connectivity. To understand the consequences of different environmental scenarios on heterogeneity and connectivity, it is necessary to understand the scale at which different environmental factors affect β-diversity. We sampled macrofauna from intertidal sites in nine estuaries from New Zealand’s North Island that represented different degrees of stress derived from land-use. We used multiple regression models to identify relationships between β-diversity and local sediment variables, factors related to the estuarine and catchment hydrodynamics and morphology and land-based stressors. At local scales, we found higher β-diversity at sites with a relatively high total richness. At larger scales, β-diversity was positively related to γ-diversity, suggesting that a large regional species pool was linked with large-scale heterogeneity in these systems. Local environmental heterogeneity influenced β-diversity at both local and regional scales, although variables at the estuarine and catchment scales were both needed to explain large scale connectivity. The estuaries expected *a priori* to be the most stressed exhibited higher variance in community dissimilarity between sites and connectivity to the estuary species pool. This suggests that connectivity and heterogeneity metrics could be used to generate early warning signals of cumulative stress.

## Introduction

The effects of spatial scale on diversity are readily apparent from species accumulation curves that are structured by increasing the sampling area or the number of habitats [1], [2]. This implies a strong interaction between the regional species pool and the local richness. β-diversity can represent the difference in species composition between local and regional assemblages [3] and in this context it reflects heterogeneity in community composition and ecological connectivity [4]–[6]. Under this definition, β-diversity has been suggested as a measure of ecosystem resilience across scales [7], where the difference between a local site’s richness and the size of the regional species pool reflects the connectivity between biological assemblages and thus influences the recovery potential of ecological systems [6], [8]. However, cumulative disturbance can cause both ecological and physical habitat fragmentation and local community homogenisation, with the consequent loss of ecological connectivity feeding back on the potential for recovery [8], [9]. Habitat homogenisation and ecological fragmentation are amongst the main threats to biodiversity [10], and β-diversity has been promoted as providing essential information for conservation management by highlighting the consequences of biodiversity loss to ecosystem resilience [6], [11], [12].

Under the current global situation of marine ecosystem degradation due to cumulative stressors [13], we need to identify and test surrogate variables that indicate the potential for dynamic changes in community state. In this context, β-diversity is essential for identifying relevant scales of change and understanding ecosystem processes related to anthropogenic activities [6], [14]. A range of natural factors have been implicated in affecting changes in β-diversity patterns, including distance between locations and mechanisms related to dispersal, local environmental variability and changes in the strength of species interaction [15]–[17]. Empirical evidence for the relative importance of these factors is inconsistent [18] and it is likely to vary over space and time scales [6]. Human activities can also have different effects on patterns in β-diversity at different scales, e.g. at local scales stress could decrease heterogeneity [19] while at regional scales it could initially increase heterogeneity through the loss of ecological connectivity, i.e. ecological fragmentation [7], [10]. Importantly, β-diversity may show non-linear responses to stress, as diffuse sources of anthropogenic disturbance can operate in a cumulative fashion, implying the importance of understanding thresholds of change in β-diversity [9], [20].

While our ability to identify abrupt regime shifts in natural systems has improved, detection of potential early-warning signals is still very limited [21]–[23]. At present, the only way of detecting a threshold is to cross it [24] and thus identifying indicators and trends that occur in advance of large shifts in ecosystem state is critical for successful management, especially if there is hysteresis in recovery dynamics [25]. Early warning signals for regime shifts would be useful for managing transitions, however, response patterns still remain ambiguous limiting application in management. While most of the proposed indicators are theoretically well developed, there have been few empirical tests [26]–[28], but see [23], [29], with effective long-term time-series monitoring required [21], [29], [30]. In this context, there is a need to understand connectivity and heterogeneity patterns before systems transition to a degraded state. However, the expected responses to disturbance may not be evident in low stress scenarios as heterogeneity between locations and habitat types would maintain the regional species pool [30]. This suggests that there is likely to be a threshold to the effects of habitat fragmentation on β-diversity, when habitat patches are too small and unconnected to sustain viable populations [2] and thus the regional species pool decreases, causing broad-scale homogeneity [10] and low β-diversity at medium to large scales. β-diversity is also a promising measure of ecosystem integrity, but its use needs to be verified by testing for scale-dependence in relation to disturbance.

The estuaries in New Zealand can be considered low stressed systems (compared to more populated regions) and we aimed to assess local and regional scales of heterogeneity and ecological connectivity in these systems. In a naturally heterogeneous estuarine system we would expect local connectivity patterns (connecting patches on the 10–100 m scale) to be strongly influenced by micro-scale environmental variability, while at larger scales we would expect estuarine morphology and hydrodynamics to increase in importance, although these different scales will interact [2], [6], [31], [32]. Diffuse sources of stress in these estuaries are mainly caused by land-based environmental stressors that include runoff from agricultural, industrial and urban land-use [10], [33], [34] modified by the size and topography of the catchment as well as by climate. Once some understanding of the scale-dependence of environmental factors that contribute to ecological connectivity and heterogeneity has been gained, exploring changes in β-diversity should provide information about the subtle effects of human disturbance in estuaries, such as changes in small-scale patchiness [10] and loss of connectivity between ecological units [6].

Here we consider three hypotheses related to spatial scale, land-use stressors and β-diversity: (1) Within-site small-scale heterogeneity in species richness primarily depends on local environmental variables. (2) The estuarine community heterogeneity and connectivity among communities (between-sites scale) is primarily controlled by large-scale changes in estuarine characteristics and the within estuary habitat variability. (3) Heterogeneity and connectivity patterns are controlled by stress derived from human uses in the estuary causing small-scale homogeneity, resulting in ecological fragmentation and consequent large-scale loss of connectivity. The alternative hypotheses imply that the heterogeneity and connectivity patterns are controlled by distance between locations that determines connectivity among communities through species’ dispersal patterns. This study employs a novel approach based on the empirical study of β-diversity patterns across scales, from local patches to regional scales, compared to most studies which focus on a single scale. Using this approach we aim to assess shifts in scale-dependent heterogeneity and connectivity in systems subjected to a gradient of diffuse source stressors.

## Materials and Methods

### Characterisation of the Estuaries

**Figure 1:**
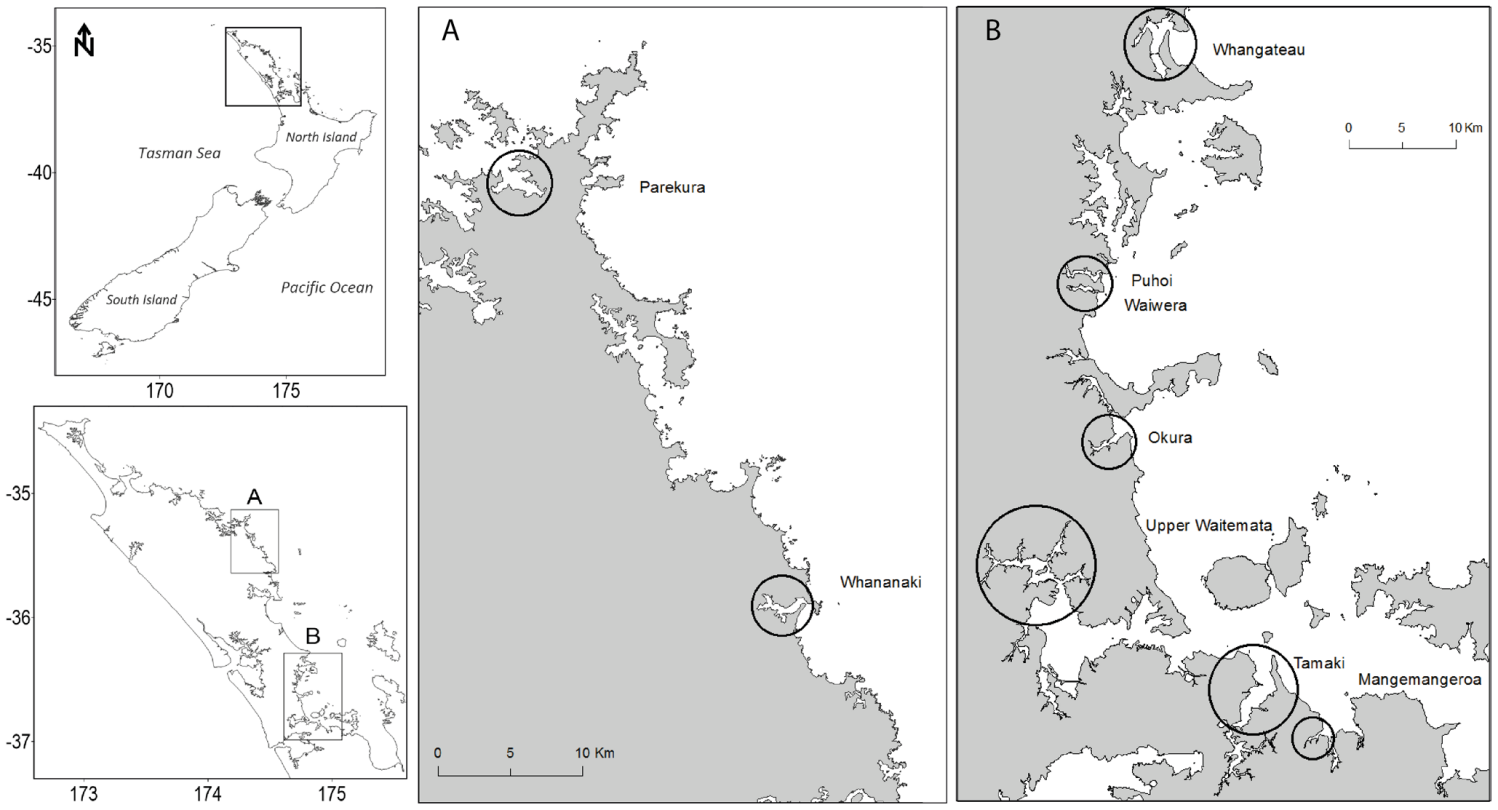
Map of the study area: 9 estuaries in New Zealand North Island.

A set of nine estuaries with different intensities of land-based stressors were selected around the North Island of New Zealand (Fig. 1): Parekura, Whananaki, Okura, Puhoi, Waiwera, Whangateau, Mangemangeroa, Tamaki and Waitemata. In order to identify variability between estuaries linked to natural or anthropogenic factors, a range of potential explanatory variables for changes in β-diversity were obtained from existing data bases and sampling. These variables covered local (site), within estuary, and estuary/catchment scales. The estuarine and catchment characteristics were available from the New Zealand Estuarine Environment Classification Database (NZEED, [35]). The percentage of land cover in the catchment of natural, pastoral and exotic (i.e. plantation forests) vegetation and urbanisation were used as surrogates for stress caused by human uses in the catchment [33], [36]. Tamaki and Waitemata estuaries were expected to be the most stressed as the percentage of urban cover was over 40%, while Parekura and Whananaki had the largest proportion of natural cover in the catchment (Table 1). The estuaries also exhibited variable morphology and hydrodynamics (Table 1), although they were all coastal-ocean dominated systems with low freshwater input relative to the tidal exchange. The Shore Complexity Index (as the length of the perimeter of the estuary shoreline divided by the circumference of a circle that has the same area as the estuary; 1 for simple and <0.1 for complex shoreline) and the Closure Index (as the width of the estuary mouth divided by the length of the perimeter of the estuary shoreline; 0.4 wide mouth to <0.01 narrow entrance), were also available from NZEED. Total nitrogen and ammoniacal-nitrogen as annual average loads to each estuary were available (http: //www.mpi.govt.nz/environment-natural-resources/water/clues), but preliminary analyses showed no effects of these parameters on the β-diversity measures and were not included further.

**Table 1:** Estuarine and catchment characteristics.

Estuaries	Estuary area	Catchment area	Shore length	SC	CI	River inflow	River discharge	Rainfall	Runoff	Tide range	Intertidal(%)	Natural(%)	Pastoral(%)	Urban(%)	Exotic(%)
Mangemangeroa	0.6	10.0	7.4	0.34	0.09	0.02	0.4	1208	300	2.4	86.9	26.6	69.9	3.4	0.09
Okura	1.4	27.4	12.7	0.31	0.04	0.06	1.2	1401	457	2.2	79.3	40.7	36.9	0.6	21.8
Parekura	3.6	23.2	14.4	0.45	0.05	0	1.2	1598	664	1.6	36.9	77.3	17.1	0	5.6
Puhoi	1.7	43.0	18.7	0.25	0	0.03	2.2	1600	764	2.1	70.6	32.8	55.5	0	11.4
Tamaki	16.9	108.8	94.6	0.15	0.02	0.004	4.1	1208	250	2.4	40.0	1.96	24.7	73.1	0.2
Waitemata	78.8	427.3	260.1	0.12	0.01	0.003	19.7	1468	430	2.3	36.2	18.6	34.5	42.6	4.2
Waiwera	1.0	37.9	11.7	0.3	0.001	0.04	1.9	1577	643	2.1	64.5	47.1	52.3	0.4	0.3
Whananaki	2.1	53.9	16.8	0.3	0.01	0.04	2.9	1731	778	1.6	75.3	65.1	34.5	0	0.2
Whangateau	7.5	42.4	31.9	0.3	0.01	0.01	2.1	1596	553	1.9	85.4	19.6	75.6	0.9	3.6

Data obtained from the New Zealand Estuarine Environment Classification Database [35].

Estuarine water area at high tide and land catchment area in km^2^. Shore length in km. The shore complexity index (SC) as the length of the perimeter of the estuary shoreline divided by the circumference of a circle that has the same area as the estuary (1 simple and <0.1 complex shoreline). The closure index (CI) as the width of the estuary mouth divided by the length of the perimeter of the estuary shoreline (0.4 wide to <0.01 narrow entrance). Annual river inflow as the ratio of river inflow to total estuary volume at high water. The mean annual discharge of river to estuary in cumecs. Mean catchment rainfall (mm/yrs) and mean annual runoff (mm/km^2^). Mean tide range in metres. Intertidal, as the percentage of intertidal area in the estuary at high water. Land-use variables based on the percentage of catchment covered by natural, pastoral and exotic vegetation and urban areas.

### Sampling Sites

The estuaries were sampled in spring-summer between 2006 and 2011: summer 2006 and 2009 in Waitemata, spring-summer 2009 and 2010 in Tamaki, spring 2011 in Mangemangeroa and summer 2008 in the other estuaries. However, for all sites not sampled in the summer of 2008 (i.e. some sites from Waitemata, Tamaki and Mangemangeroa), time series of macrofaunal and sediment composition at most sites within the estuary were available over a 5 year period and multivariate analysis of these variables did not show a strong annual variation relative to within or between estuarine differences. For example, 7 of the 10 sites in Mangemangeroa had been sampled in every year since 2005 in early spring and late summer and we tested that differences in community composition based on presence/absence data between these 7 sites did not differ between the 2011 year and the others, and that late summer sampling was not different to early spring.

Between 9 and 10 randomly located intertidal sandy sites were sampled in each estuary, with the exception of Okura, 8 sites, Whananaki, 6 sites, and Tamaki, 7 sites. Ten core samples of 13 cm of diameter and 15 cm depth were obtained at each site (with the exception of Mangemangeroa where only 6 samples were available). This sampling strategy covered the within-patch heterogeneity with 6–10 replicates obtained at each site (site area c 50 m^2^), and the within estuary heterogeneity, by sampling 10 sites across sand-flats with variable sand-mud content. Distance between sites varied from an average of 15.7 km in Waitemata to 0.4 km in Mangemangeroa (Appendix 1), related to the size of the estuary.

Core samples were sieved through a 500 µm mesh and the retained organisms were identified to the lowest practical taxonomical level (generally species). Small cores (2 cm depth) were used to sample surface sediment for grain size, organic content and benthic chlorophyll *a* at each site (organic content was not available for Waitemata and Tamaki and chlorophyll *a* was not available for Mangemangeroa and Tamaki). The presence of shell hash was assessed at each site, based on visual assessment of shell material present on the sediment surface within 5 0.25 m^2^ quadrat photos, as: no shell (0%), rare (0–5%) and presence when there was a moderate to high abundance (>5% and it was rarely higher than 40%).

No specific permissions were required for sampling these locations as our sampling is a permitted activity. Field studies did not involve endangered or protected species.

### β-diversity Measures

Numerous studies have addressed the importance of β-diversity to ecological patterns by using a great variety of metrics; a consensus has not been reached and variety of metrics prevail [3], [37]. In this work we explored 2 different measures: one strictly related to species accumulations in space (additive β-diversity [38]); and one related to community heterogeneity (Jaccard’s dissimilarity [4]). Additive β-diversity (γ-α) measures heterogeneity from local to larger scales and can be adapted to measure connectivity between locations [6], [39], [40], with its magnitude dependent on the both α- and γ-diversities. We used additive β-diversity (γ-α) instead of the multiplicative β-diversity (γ/α) [41], as we consider it better represents the connectivity between local and regional species richness. Preliminary analysis with both measures proved that general connectivity patterns were consistent across both measures.

We adopted a consistent and hierarchical approach in order to assess β-diversity at different spatial scales, from sampling site to estuary. Within-site heterogeneity of species richness, hereafter β-site, was considered as the difference between average species richness (α-site diversity) and total species richness (γ-site diversity). The α-site diversity was the average of species richness from the 10 replicates in each site and the γ-site was estimated as the species richness obtained from the random accumulation of 10 replicates in each site [42]. Therefore, the difference between the average number of species from the 10 replicates and the total number of species recorded in a site reflected the heterogeneity at the within-site patch scale [10]. Within-estuary connectivity of species richness, β-conn, was estimated for each site from the difference between γ-diversity at the estuary scale (γ-estuary diversity) and the total species richness at a site, γ-site, where the γ-estuary diversity was the species richness predicted to be obtained from the random accumulation of the 10 replicates at 10 sites in each estuary. Within-estuary and between-estuaries heterogeneity in communities was assessed by calculating the Jaccard’s dissimilarity index (based on species presence-absence data) for each pair of sites within each estuary and for pairs of estuaries, based on site and estuary average species composition respectively. Diversity measures across scales are summarised in Appendix 1.

### Data Analysis

Principal Components analysis was done to obtain an ordination of the estuaries based on the morphological, hydrodynamic variables and land-use in the catchment (variables included in Table 1). Spearman rank correlation tested the link between the different diversity scales, from α- to β- and γ-diversities. Kruskal-Wallis tests were performed to assess the significance of differences between β-diversity measures across estuaries.

Aiming to understand the scales of factors controlling average and total site species richness (α- and γ-site), within-site heterogeneity (β-site), and site connectivity to the estuarine species pool (β-conn), we performed Random Forest (RF) and Generalised Additive Models (GAM). These statistical tools are particularly useful for considering a large number of interdependent variables, as they are flexible regarding missing data and allow the inclusion of complex non-linear interactions that usually exist in natural communities [43]. The aim of combining these two approaches was to take advantage of their individual strengths, e.g. inclusion of a large number of variables in RF, with the subsequent identification of the most important set of variables to perform a GAM that allows the inclusion of non-linear effects. RF is a machine learning based approach that uses a regression tree approach to recursively partition predictor variables. Bootstrap samples are drawn to construct multiple trees and each tree is grown with a randomized subset of predictors [44]. In this work, RF were built using 500 regression trees to model relationships between the diversity measures and environmental data. The number of predictors to be chosen randomly at each split was set as one-third the number of variables. The GAM models were adjusted to a quasi-Poisson distribution of the data. The most parsimonious models were identified by backwards selection using the GCV score [45].

The RF and GAM analyses included the effects of the environmental variables at different scales: estuarine and catchment variables (estuary and catchment areas, shore length, complexity and closure indices, river inflow and discharge, mean rainfall and runoff, tide range, percentage of intertidal area and land-based stressors estimated from the percentages of natural, pastoral, exotic and urban cover of the catchment; Table 1) and local variables (percentages of mud, coarse sediment, medium grain and fine grain sand, organic content, shell hash and chlorophyll *a*, Table 2). The distance of each site from the estuary mouth was also included in the analysis. Prior to the analysis, we checked possible significant inter-correlations and co-variances between the environmental variables and found high Spearman correlation (at >0.8 and with high covariance correlation considered at >0.70) between fine sand and mud and fine sand and intertidal area; shore length, catchment area and discharge were highly correlated amongst themselves and with estuarine area, exotic coverage and complexity index. The variables fine sand, shore length, catchment area, and river discharge were excluded from the analysis.

**Table 2:** Ranges of the sediment variables across sites.

Estuaries	% Coarse	% Sand	% Mud	Organic	Chl. *a*
Mangemangeroa	0.1–5.3	0.3–34.6	0.6–37.1	1.3–5.4	−
Okura	0.1–15.1	0.2–2.6	4.3–32.3	0.9–2.3	3.3–9.9
Parekura	0.9–47.9	1.5–24.9	3–64.3	1–5.8	2.4–11.8
Puhoi	0.1–1.4	0.3–22.9	3–27.1	1.6–3.2	4.8–7.9
Tamaki	0.5–8	0.9–73.8	7.3–79.4	−	−
Waitemata	0–9	0.3–22.7	10.2–86.7	−	8.8–32.1
Waiwera	0.05–4.6	0.4–25.1	0.1–65.3	1.2–3.3	4–9.2
Whananaki	0.6–9.8	1.5–2.7	4.1–23.1	1.7–2.9	5.5–9.5
Whangateau	0.1–3.7	11.2–35.3	1.2–24.3	0.6–3.6	4.5–14.6

Percentages of coarse sediment, medium-grain sand and mud, organic content and chlorophyll *a*.

Mantel’s tests were performed to assess the correlation between Jaccard dissimilarity and spatial or environmental distances at 2 spatial scales (between sites and between estuaries). For spatial distances, both the distance between the estuaries (coordinates in the middle of estuary mouth) and distances between sites within the estuaries were calculated. For the environmental distances, Euclidean distances were obtained between sites based on the local environmental variables (sediment composition, organic content and chlorophyll *a*) and between estuaries based on the estuarine and catchment variables (data included in Table 1). In order to determine the role that spatial and environmental factors played in driving connectivity, Mantel tests were also performed to assess the significance of the correlations between pairs of matrices of spatial and environmental distance and differences in β-conn between sites in each estuary.

Statistical analyses were performed with the R program, v.2.11.0. The similarity measures were obtained with PRIMER statistical package [46] and species accumulation curves were obtained with EstimateS [47].

## Results

### Estuaries Characterisation

**Figure 2:**
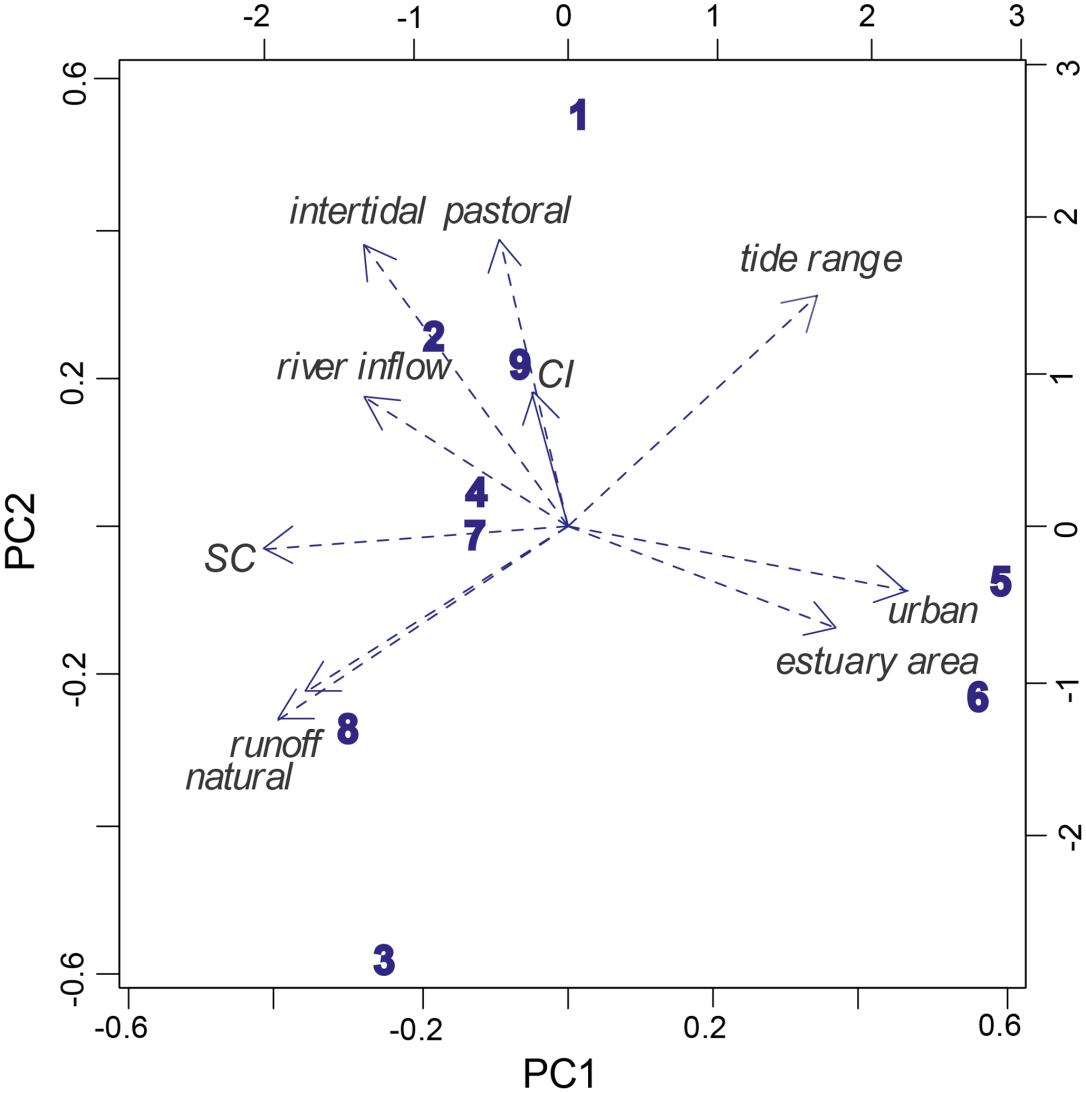
Principal Components of the estuarine and catchment environment data (arrows; data from Table 1) at each estuary: 1, Mangemangeroa; 2, Okura; 3, Parekura; 4, Puhoi; 5, Tamaki; 6, Waitemata; 7, Waiwera; 8, Whananaki; 9, Whangateau. PC1∶41% and PC2∶22% of explained variance.

The Principal Components analysis explained 78% of the variance between estuaries with 3 axes related to land-use activities, estuarine morphology and hydrodynamics (Table 1). The first two axes explained >50% and were related to most of the variables (Fig. 2), although the estuary closure (CI) and shore complexity (SC) indices were more closely related to the third axis. Tamaki and Waitemata were the most urbanised estuaries (73 and 43% of the catchment area was urbanised, respectively; Table 1); Whangateau and Mangemangeroa had a high pastoral cover in the catchment (76 and 70% respectively); Okura, Puhoi and Waiwera had approximately half pastoral and half natural cover, and Okura and Puhoi were also characterised by presence of exotic vegetation, 22 and 11% respectively (this factor was mainly related to the fourth axis); Whananaki had higher natural (65%) than pastoral cover and the catchment in Parekura was mostly covered by natural vegetation (77%).

Regarding the environmental variables, Whananaki was characterised by the highest mean runoff, with an annual average of 778 mm/km^2^; Okura, Puhoi and Waiwera were characterised by a relatively high river inflow (0.03–0.06 annual ratio of river inflow); Waitemata (79 km^2^) and Tamaki (17 km^2^) had the largest estuarine area; while Mangemangeroa (87%) and Whangateau (85%) had the highest percentage of intertidal area. Waitemata and Tamaki had the most complex shorelines (with multiple arms, SC<0.2), while the simplest shores were found in Parekura and Mangemangeroa (SC = 0.45 and 0.34). Mangemangeroa and Parekura were the most open estuaries (CI = 0.09 and 0.05 respectively), whereas the estuary mouth at Waiwera and Puhoi had narrow entrances (CI<0.001) (Fig. 2). The estuaries also varied in sediment composition, chlorophyll *a* and organic content across sites (Table 2).

### Patterns of Diversity at the Local Scale

**Figure 3:**
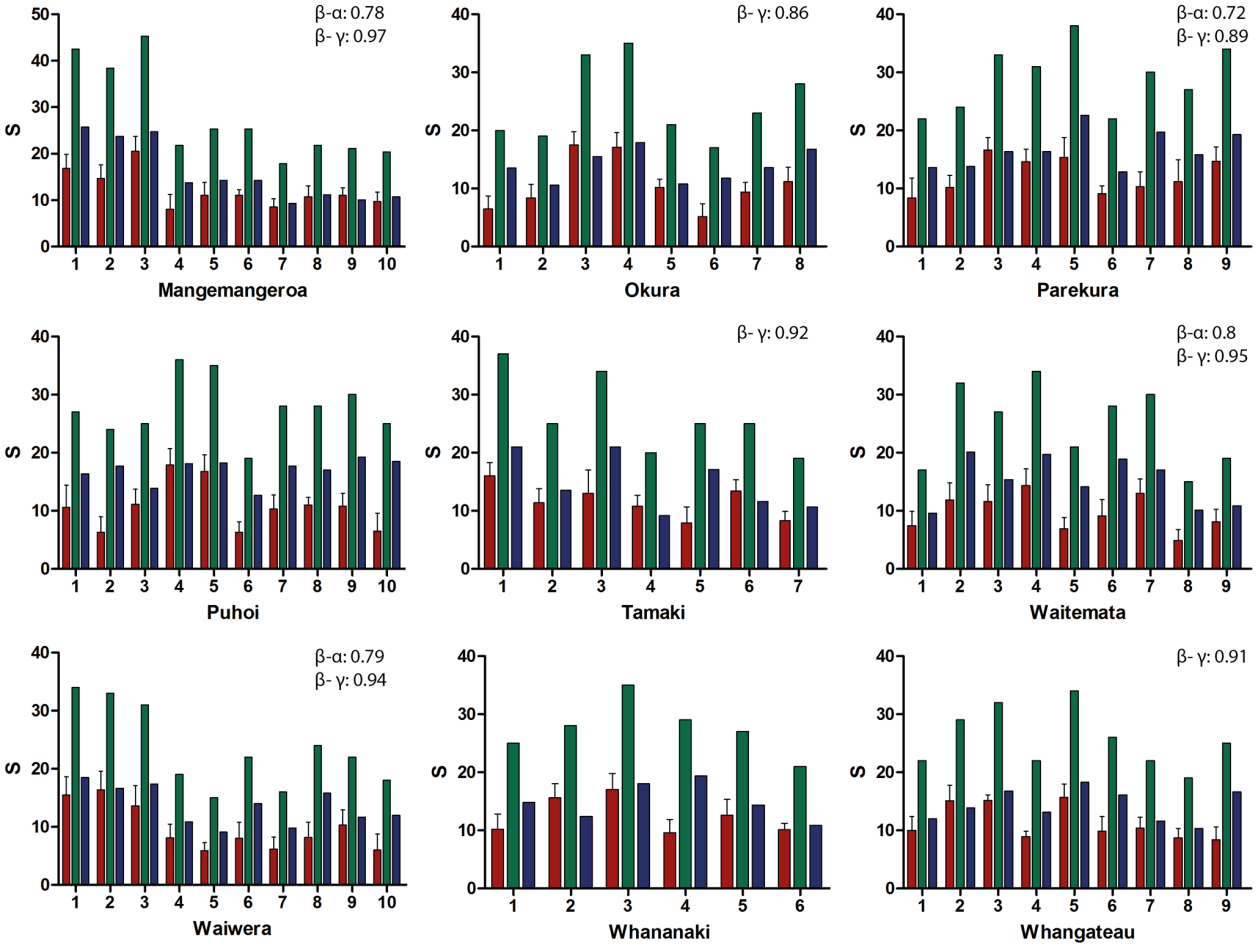
Diversity measures at the site scale in each estuary: α-site (light grey bars, mean and SD), γ- site (black bars) and β-site (grey bars). Sites: 1 to 10. Significant Spearman correlation (ρ) between diversity measures at each estuary is included in left corner of graphs.

α-site diversity was generally highly variable within the sand flats of each estuary and ranged from an average of 5 to 20 species per site (Fig. 3). The γ-site was positively related with α-site diversity. β-site diversity was also variable within estuaries and was more highly correlated with γ-site than to α-site for each estuary (Fig. 3); overall values across estuaries: ρ 0.9, p<0.001 and 0.6, p<0.001 for β-site and γ-site or α-diversity respectively. The Kruskal-Wallis test for the comparison of β-site across estuaries was not significant.

**Figure 4:**
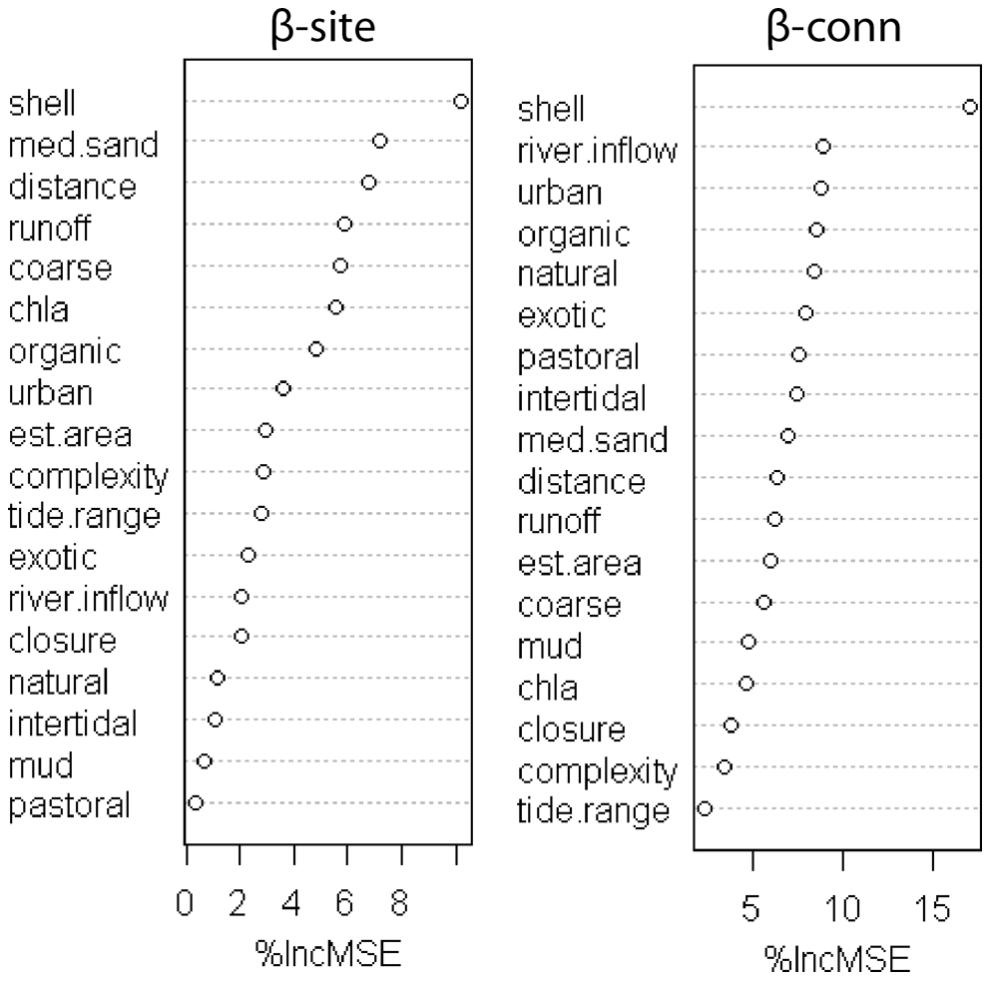
Importance of the explanatory variables in the Random Forest models for β-site and β-conn measures.

The RF and GAM models for the local diversity measures explained 21 to 51% of the variance (Table 3), with the GAM models consistently explaining a higher proportion of the variance. Shell hash had positive effects on α-, β- and γ-site diversities and a non-additive linear interaction of chlorophyll *a* and mud had negative effects on α-site. In most cases, the percentage of medium-grain sand was a good surrogate for the shell hash, but the percentage of explained variance was generally lower for this variable. For β-site diversity the most important variables were shell hash and medium sand, with positive effects, and to a lesser extent the distance to the estuary mouth, with negative effects, and chlorophyll *a* with non-linear effects. The catchment variables river runoff and exotic cover had positive effects on β-site (Fig. 4).

**Table 3:** Summary of the Random Forest (RF) and General Additive Model (GAM) for the diversity measures.

Diversity measures	% RF	%GAM	Local factors		Estuarine/catchment factors	
			RF	GAM	RF	GAM
α-	30	51	shell	+shell-p***, −chl.*a*: mud*	ns	ns
γ-site	35	52	shell, med.sand	+shell-p***, +shell-r*	ns	ns
β-site	21	51	shell, med.sand, distance	+shell-p***, +shell-r**, s(chl.*a*)**	ns	+runoff*, +exotic*
β-conn	46	59	shell	−shell-p***, −shell-r**, −coarse*	inflow, urban	−inflow***, +intertidal**_,_ −runoff^.^

Percentage of variance explained by each model and the significant effects of the variables at local and estuarine/catchment scales. All the GAMs were significant at p<0.001. Factor shell in the GAM is included as a categorical variable: “shell-p”, presence of shell, and “shell-r”, rare shell content.

*** p<0.001,

** p<0.01,

* p<0.05 and.: p<0.01.

“ns”: non-significant effects. +/− indicates the direction of the effects. s(factor) indicates smooth effects. “: ”crossed effects interaction.

### Patterns of Connectivity at the Estuary Scale

**Figure 5:**
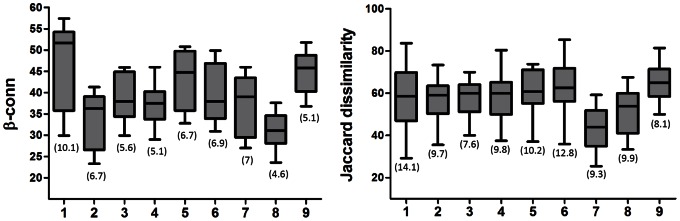
Box plots for β-conn and between-sites Jaccard’s dissimilarity in each estuary: 1, Mangemangeroa; 2, Okura; 3, Parekura; 4, Puhoi; 5, Tamaki; 6, Waitemata; 7, Waiwera; 8, Whananaki; 9, Whangateau. Boxplots include median, 25 and 75 percentiles and maximum and minimum values. In brackets the standard deviation of the β-diversities across each estuary.

**Figure 6:**
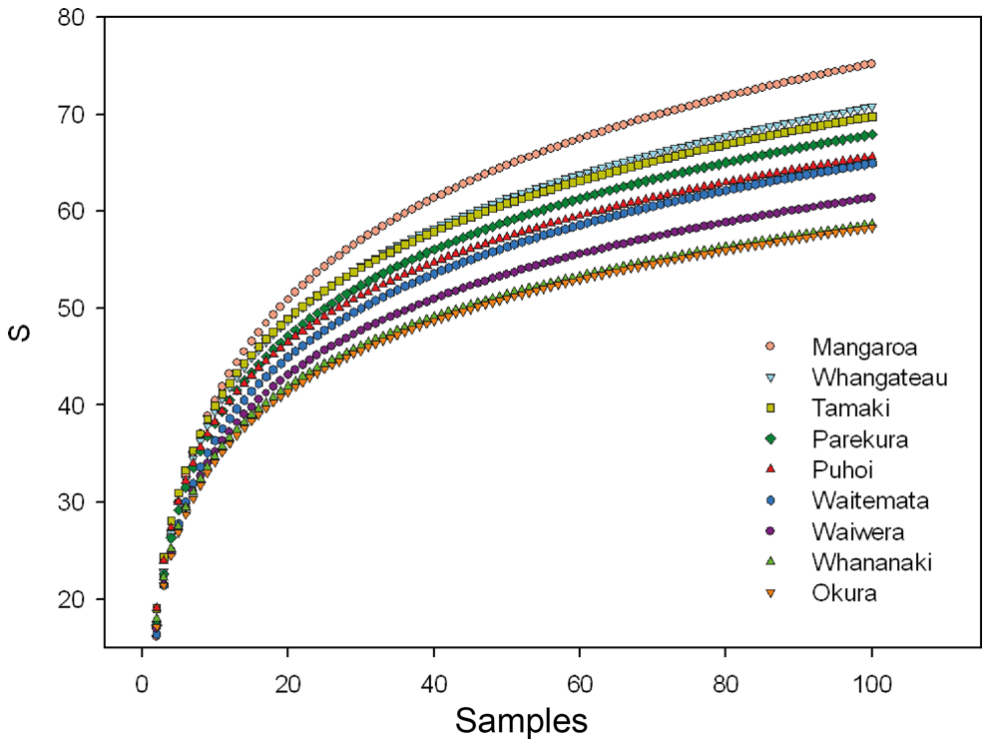
γ-estuary, as the species accumulation at 100 samples in each estuary; 10 sites and 10 replicates per site.

The average β-conn (Fig. 5) was higher in the estuaries with high γ-estuary (Fig. 6), Spearman ρ 0.49, p<0.001. Differences in β-conn across estuaries were significant for Whananaki compared to Whangateau and Mangemangeroa (p<0.001 for Kruskal-Wallis test). Within each estuary, the β-conn was negatively linked to γ-site (Spearman ρ −0.78, p<0.001), i.e. lower β-conn in the sites with highest total richness, and the highest variations within an estuary occurred in Mangemangeroa, Waiwera and Waitemata (Fig. 5). Differences in β-conn between all pairs of sites within an estuary were not related to spatial distance between sites for any estuary but were related to environmental distances for Tamaki, Waitemata and Waiwera (although only weakly related for the latter, Table 4).

**Table 4:** Summary of the Mantel test.

Matrices comparison		Estuaries	Mantel test r^2^	p-value
Within estuaries	β-conn-environmental	Tamaki	0.6	0.03
		Waitemata	0.5	<0.01
		Waiwera	0.3	0.04
	Jaccard-spatial	Parekura	0.4	0.02
		Puhoi	0.4	0.01
		Tamaki	0.6	<0.01
	Jaccard-environmental	Parekura	0.4	0.02
		Puhoi	0.6	<0.01
		Tamaki	0.7	0.02
		Waitemata	0.5	0.01
		Whangateau	0.5	<0.01
Between estuaries	Jaccard-environmental	all	0.6	0.02

Significant results for the correlation between matrices of β-diversity (β-conn and Jaccard’s dissimilarity) with spatial (geographic distance) and environmental (Euclidean distance) data. For Jaccard’s dissimilarity the Mantel’s comparisons were done for matrices between pair of sites within each estuary and also between estuaries.

In comparison with local (site) diversity measures, the predictive models for β-conn included more variables at the estuary scale. However, β-conn was also negatively related with coarse sediment and the presence of shell hash (Table 3), reflecting the link with site richness (γ-site). River inflow had negative effects and the intertidal area had positive effects on β-conn. The RF also included urban cover as the third most important variable for explaining variability in β-conn (Fig. 4). The estuaries Mangemangeroa and Whangateau, with large intertidal areas, had the highest γ-estuary, and Waiwera, Whananaki and Okura, with high river inflow and runoff, had the lowest γ-estuary diversity (Fig. 6). These morphological and hydrodynamic differences between estuaries were reflected in the models for β-conn (Table 3).

### Dissimilarity Patterns at Local and Regional Scales

Jaccard dissimilarity between sites (Fig. 5) was lowest in Waiwera (p<0.001 for Kruskal-Wallis test) and Whananaki (non-significant). In the other estuaries the average values were similar and the highest within-estuary variation occurred in Mangemangeroa and Waitemata. The dissimilarity between sites was significantly correlated with the spatial and environmental distances in Parekura, Puhoi and Tamaki, but just environmental distance in Waitemata and Whangateau (Table 4).

Jaccard dissimilarity between estuaries was not significantly correlated with the spatial distance between estuaries, but it was correlated with the environmental distance based on estuarine/catchment variables (Table 4).

## Discussion

**Figure 7:**
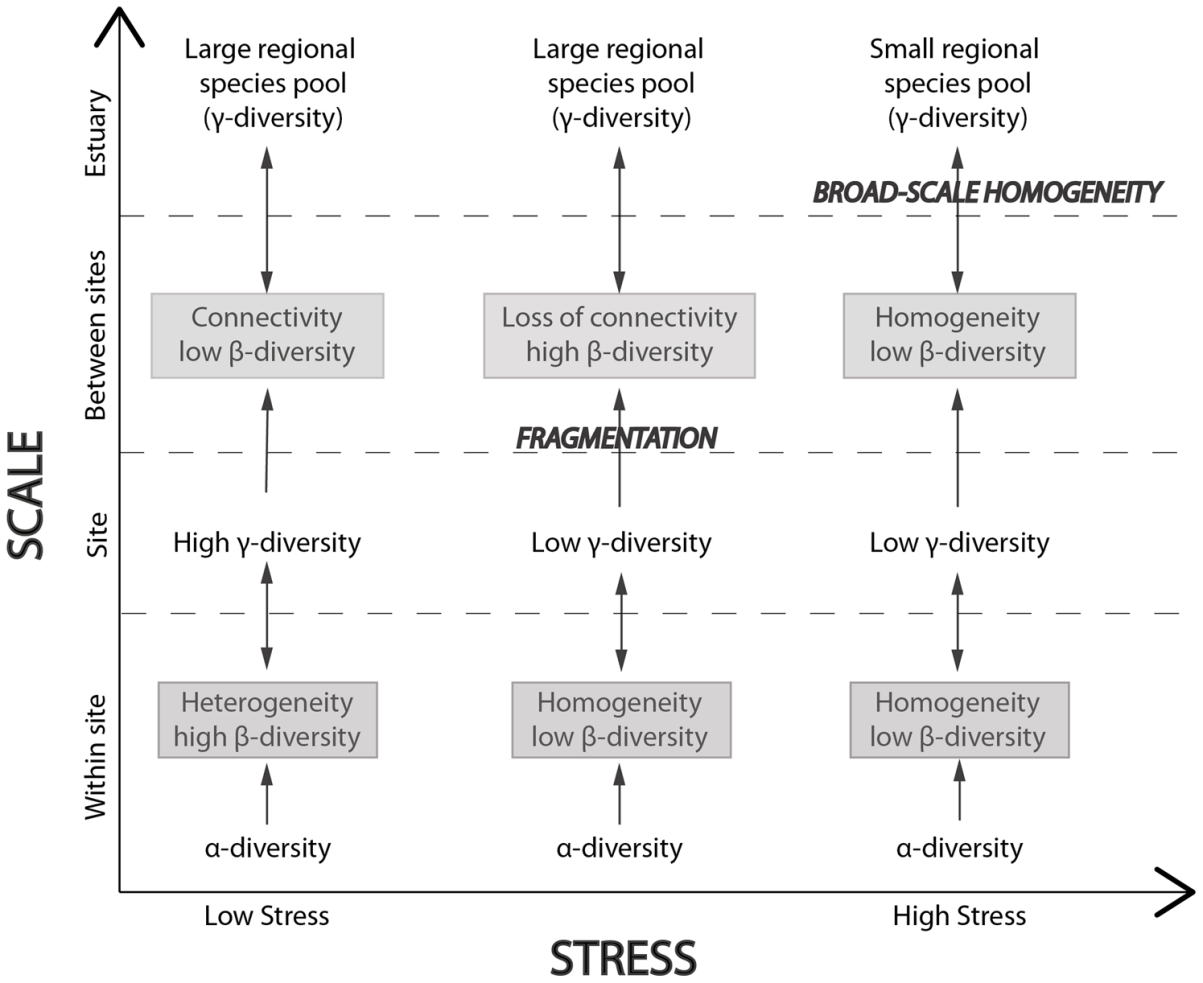
Predicted changes of β-diversity, representing heterogeneity and connectivity measures across scales, under different stress scenarios.

Land-based environmental stressors often cause small but cumulative impacts on estuarine ecosystems [30]. The principal source of stress in New Zealand intertidal areas is the input of land-based sediments caused by the reduction of native vegetation in the catchment. Under this stressor, intertidal communities undergo loss of small-scale heterogeneity and large-scale connectivity [34]. We predicted that the “no-stress” situation in intertidal sand-flats would be represented by large estuarine species pools (γ-estuary) but also by high total species richness at the site scale (γ-site), as a product of high within-site heterogeneity (β-site), with good connectivity (low β-conn) (Fig. 7) [6], [10]. In stressed systems we would expect reduced β-site, and habitat fragmentation at the estuary scale to reduce connectivity leading to high β-conn [30], [48]. If stressors persist at our sites, we would then expect that over time the positive relationship between β-conn and the stressor would disappear, then reverse, accompanied by a sharp decrease in β-conn at the estuary scale (Fig. 7) due to broad-scale homogenisation that would cause a significant reduction of species richness at the estuary scale [10].

AS we aimed to assess the effects of stress on β-diversity, across scales, before a degraded state with changes in heterogeneity and connectivity, the nine estuaries included in our study represent different but relatively low stress conditions: Waitemata and Tamaki were the most urbanised estuaries and Parekura and Whananaki had the highest extension of natural cover in the catchment. However, heterogeneity and connectivity within an estuary also depends on natural factors, such as currents and dispersion barriers [7], [32], [49], and the nine estuaries also differed in their morphological and hydrodynamic characteristics. The dissimilarity in communities between estuaries we observed was related to their morphology, hydrodynamics and land-use and not to spatial distance. Within estuaries, environmental variation was important in explaining patterns of community heterogeneity (Jaccard dissimilarity) for half the estuaries but it was only important in explaining differences in connectivity (β-conn) for one third of the estuaries. The distance between sites was not important in explaining within-estuary heterogeneity and connectivity, which again reflected the importance of environmental variability rather than the spatial distance between locations.

Local-scale β-diversity (β-site) was strongly related to local-scale γ-diversity in all estuaries, rather than local-scale α-diversity, and it was predicted by both local-scale and estuary-scale environmental factors. Similarly, estuary-scale β-conn was predicted by local-scale and estuary-scale environmental factors and it was related to γ-diversity of the estuary, while it had a negative relationship with γ-diversity at the local scale, causing within-estuary variability in connectivity. The dissimilarity between sites was most highly variable in Mangemangeroa, which translated into higher community heterogeneity within the estuary. This probably contributed to a high average and variance in β-conn and to this estuary having the largest regional species pool. Conversely, the sites in Whananaki and Waiwera estuaries, with the lowest regional species pool, had low between-sites dissimilarity and Whananaki had the highest within-estuary connectivity.

The importance of local factors in explaining β-diversity continued as scale increased to the estuary scale. The most consistently selected predictor variable was shell material, which was positively related to α-, β- and γ-diversities at the local scale and negatively related to estuarine connectivity, β-conn. Shell material has previously been found to increase both average species richness and within-site heterogeneity [19]. Only for β-site was a non-sediment related variable important (distance to mouth). In our study, muddy sites were generally located in the upper estuary nearer to sources of sediment run-off, and were more homogeneous than areas where sedimentation rates are lower [34]. Unexplained variability ranged from 41 to 79%, indicating that the variability in species diversity, particularly at local scales, was influenced by other factors, e.g. small-scale biological disturbance, interaction between species, competition for resources [50], [51]. Interestingly, while the measures selected as predictors of γ- and α-site diversities were all local-scale variables, models predicting β-diversity at both the local and within-estuary scales also selected estuary-scale variables. These included intertidal area, river inflow, runoff and urban or exotic cover. There is a certain degree of correlation between these last three variables, with runoff being highest in estuaries with extensive urban areas (like Tamaki and Waitemata). River inflow also represented a group of relatively small estuaries, with river-type morphology and low stress (Okura, Puhoi, Waiwera and Whananaki). The percentage of intertidal area was highest in the estuaries with the largest estuarine species pool (Mangemangeroa and Whangateau), both open estuaries with high coverage of pastoral land. Natural variability between locations could mask expected responses to stress and the control of large-scale variables over the β-diversities could be in part related to the differentiation of estuarine groups; e.g. the exotic cover and runoff, which are expected to promote habitat homogeneity, were positively correlated with β-site.

While we should treat the importance of the selected predictors cautiously, due both to potential correlations and the small number of estuaries (9) sampled, the predictors did include those representing both geomorphology and stress. At the within-estuary scale, connectivity (β-conn) was negatively related to a potential stressor (runoff), while at the site scale, heterogeneity (β-site) was positively related to local variables related to habitat heterogeneity (shell hash). The average within-site heterogeneity in species richness (β-site) was similar across estuaries, but the estuary heterogeneity in species composition (similarity between sites) was lowest in Waiwera and site-connectivity to the regional species pool (β-conn) was lowest in Mangemangeroa and Whangateau. These patterns overlapped with the effects of estuarine morphology and hydrodynamics on β-diversity and no strong evidence of the predicted responses to the stress gradient were identified. In systems subjected to low but cumulative disturbance, we should expect increasing variability in local species richness across space due to localised responses to disturbance [10], [26]. An early warning in our low stress scenarios would be high variability in connectivity within an estuary (β-conn), with some sites starting to lose connectivity across the estuary. The highest variation for β-conn measures occurred in Mangemangeroa, Waitemata and Waiwera. Waitemata had a high degree of urbanisation; Mangemangeroa had been undergoing changes in land-use associated with urbanisation; and Waiwera had considerable road construction across the upper estuary. Another early warning signal could be related to the heterogeneity in species composition between sites across an estuary. The highest variation for between-sites dissimilarity was again observed in Mangemangeroa and Waitemata, and in the most urbanised estuary, Tamaki. Note that Mangemangeroa had the lowest estuary connectivity (high β-conn) and Waiwera had the highest within-estuary homogeneity (low Jaccard dissimilarity).

Data from low stressed systems is needed to identify early warning signals of increasing marine degradation [13]. Most studies on early warning signals focus on temporal trends, for which long-term data bases are necessary, or data needs to be extracted from models [23], [52], [53]. Early signals based on data analysed across spatial scales in low stress systems could thus prove particularly useful tools to anticipating changes in benthic ecosystems subjected to diffuse sources of cumulative stress. The estuaries included in this study were subjected to low stress, where impact responses related to heterogeneity and connectivity measures were not strong. Results confirm the complexity of natural systems, where responses to cumulative stress in terms of shifts in heterogeneity and connectivity do not follow linear patterns and should be placed within the wider context of diversity estimates across scales (Fig. 7). However, our results suggested links between predicted land-based stressors and β-diversity, as the estuaries with larger urbanised areas in the catchment had increased variability in β-diversity across the estuary. The implications of this for management are important in the context of spatial planning and maintaining the adaptive capacity of estuarine ecosystems. While large-scale heterogeneity might still sustain a large regional species pool in the studied systems, the increased variability across some of the most stressed estuaries implies that persisting cumulative impacts could trigger ecological fragmentation across the estuary and consequent decrease of resilience of ecological communities. While this study does not suggest a single indicator of ecosystems to stress, it suggests that combined β-diversity metrics are good candidates for early warnings in low stressed systems to predict loss of resilience due to community homogenisation and loss of ecological connectivity.

## Supporting Information

Table S1 **Summary of the diversity measures from within-site to estuary spatial scales.** α-site is the average species richness at a site (mean ± SD, n = 10 replicates); γ-site is the total species richness predicted from the accumulation of 10 replicates per site; β-site is the additive β-diversity at the within-site scale (γ-site - α-site); γ-estuary is the total species richness predicted from the accumulation of 10 replicates ×10 sites in each estuary; β-conn is the additive β-diversity at the within-estuary scale (γ-estuary - γ-site); between-site Jaccard is the average similarity between all pair of sites within the estuary.
(DOCX)
