# Correction: Counting on β-Diversity to Safeguard the Resilience of Estuaries

**DOI:** 10.1371/annotation/a16aaf48-e86f-4698-8998-81d47dc0dc94

**Published:** 2013-11-07

**Authors:** Silvia de Juan, Simon F. Thrush, Judi E. Hewitt

The Figure 3 caption does not include the correct legend colors. The corrected Figure 3 caption reads:

Figure 3. Diversity measures at the site scale in each estuary: α-site (red bars, mean and SD), γ- site (green bars) and β-site (blue bars). Sites: 1 to 10. Significant Spearman correlation (r) between diversity measures at each estuary is included in left corner of graphs. doi:10.1371/journal.pone.0065575.g003 

