# Supplementary material for: Counting on β-Diversity to Safeguard the Resilience of Estuaries
Source: PLoS One. 2013 Jun 5;8(6):e65575. doi: 10.1371/journal.pone.0065575 (PMC3673921; doi:10.1371/journal.pone.0065575)
Supplement: Table S1 — Summary of the diversity measures from within-site to estuary spatial scales. α-site is the average species richness at a site (mean ± SD, n = 10 replicates); γ-site is the total species richness predicted from the accumulation of 10 replicates per site; β-site is the additive β-diversity at the within-site scale (γ-site - α-site); γ-estuary is the total species richness predicted from the accumulation of 10 replicates ×10 sites in each estuary; β-conn is the additive β-diversity at the within-estuary scale (γ-estuary - γ-site); between-site Jaccard is the average similarity between all pair of sites within the estuary. (DOCX) [file pone.0065575.s001.docx]

**Table S1** –

| Estuaries | Mean km between sites | sites | α-site | β-site | γ-site | β-conn. | γ-estuary | Between-site Jaccard |
| --- | --- | --- | --- | --- | --- | --- | --- | --- |
| Mangemangeroa | 0.4 | 1 | 16.8±3.1 | 25.7 | 43 | 32.7 | 75.16 | 42.2±14.1 |
|  |  | 2 | 14.7±2.9 | 23.7 | 38 | 36.8 |  |  |
|  |  | 3 | 20.5±3.2 | 24.8 | 45 | 29.9 |  |  |
|  |  | 4 | 8±3.2 | 13.8 | 22 | 53.4 |  |  |
|  |  | 5 | 11±2.8 | 14.3 | 25 | 49.9 |  |  |
|  |  | 6 | 11±1.3 | 14.3 | 25 | 49.9 |  |  |
|  |  | 7 | 8.5±1.8 | 9.3 | 18 | 57.4 |  |  |
|  |  | 8 | 10.7±2.4 | 11.2 | 22 | 53.4 |  |  |
|  |  | 9 | 11±1.7 | 10.1 | 21 | 54.1 |  |  |
|  |  | 10 | 9.7±2.1 | 10.7 | 20 | 54.8 |  |  |
| Okura | 1.2 | 1 | 6.5±2.2 | 13.5 | 20 | 38.3 | 58.4 | 43.7±9.7 |
|  |  | 2 | 8.4±2.3 | 10.6 | 19 | 39.3 |  |  |
|  |  | 3 | 17.5±2.3 | 15.5 | 33 | 25.3 |  |  |
|  |  | 4 | 17.1±2.5 | 17.9 | 35 | 23.3 |  |  |
|  |  | 5 | 10.2±1.4 | 10.8 | 21 | 37.3 |  |  |
|  |  | 6 | 5.2±2.2 | 11.8 | 17 | 41.3 |  |  |
|  |  | 7 | 9.4±1.6 | 13.6 | 23 | 35.3 |  |  |
|  |  | 8 | 11.2±2.5 | 16.8 | 28 | 30.3 |  |  |
| Parekura | 2.3 | 1 | 8.4±3.4 | 13.6 | 22 | 45.9 | 67.9 | 42±7.6 |
|  |  | 2 | 10.2±2.1 | 13.8 | 24 | 43.9 |  |  |
|  |  | 3 | 16.6±2.2 | 16.4 | 33 | 34.9 |  |  |
|  |  | 4 | 14.6±2.2 | 16.4 | 31 | 36.9 |  |  |
|  |  | 5 | 15.4±3.4 | 22.6 | 38 | 29.9 |  |  |
|  |  | 6 | 9.1±1.4 | 19.7 | 22 | 45.9 |  |  |
|  |  | 7 | 10.3±2.6 | 12.9 | 30 | 37.9 |  |  |
|  |  | 8 | 11.2±3.8 | 15.8 | 27 | 40.9 |  |  |
|  |  | 9 | 14.7±2.5 | 19.3 | 34 | 33.9 |  |  |
| Puhoi | 1.4 | 1 | 10.6±3.8 | 16.4 | 27 | 38.0 | 65 | 41.7±9.8 |
|  |  | 2 | 6.3±2.7 | 17.7 | 24 | 41.0 |  |  |
|  |  | 3 | 11.1±2.6 | 13.9 | 25 | 40.0 |  |  |
|  |  | 4 | 17.9±2.8 | 18.1 | 36 | 29.0 |  |  |
|  |  | 5 | 16.8±2.9 | 18.2 | 35 | 30.0 |  |  |
|  |  | 6 | 6.3±1.8 | 12.7 | 19 | 46.0 |  |  |
|  |  | 7 | 10.3±2.5 | 17.7 | 28 | 37.0 |  |  |
|  |  | 8 | 11±1.3 | 17.0 | 28 | 37.0 |  |  |
|  |  | 9 | 10.8±2.2 | 19.2 | 30 | 35.0 |  |  |
|  |  | 10 | 6.5±3.1 | 18.5 | 25 | 40.0 |  |  |
| Tamaki | 7.9 | 1 | 16±2.3 | 21.0 | 37 | 32.8 | 69.8 | 39.1±10.2 |
|  |  | 2 | 11.3±2.4 | 13.6 | 25 | 44.8 |  |  |
|  |  | 3 | 13±4.0 | 21.0 | 34 | 35.8 |  |  |
|  |  | 4 | 10.8±1.9 | 9.2 | 20 | 49.8 |  |  |
|  |  | 5 | 7.9±2.8 | 17.1 | 25 | 44.8 |  |  |
|  |  | 6 | 13.4±2 | 11.6 | 25 | 44.8 |  |  |
|  |  | 7 | 8.3±1.6 | 10.7 | 19 | 50.8 |  |  |
| Waitemata | 15.7 | 1 | 7.4±2.5 | 9.6 | 17 | 47.9 | 64.9 | 36.1±12.8 |
|  |  | 2 | 11.4±2.9 | 20.1 | 32 | 32.9 |  |  |
|  |  | 3 | 11.6±2.9 | 15.4 | 27 | 37.9 |  |  |
|  |  | 4 | 14.3±2.9 | 19.7 | 34 | 30.9 |  |  |
|  |  | 5 | 6.9±1.9 | 14.1 | 21 | 36.9 |  |  |
|  |  | 6 | 9.1±2.8 | 18.9 | 28 | 43.9 |  |  |
|  |  | 7 | 13±2.5 | 17.0 | 30 | 34.9 |  |  |
|  |  | 8 | 4.9±1.9 | 10.1 | 15 | 49.9 |  |  |
|  |  | 9 | 8.1±2.2 | 10.9 | 19 | 45.9 |  |  |
| Waiwera | 0.6 | 1 | 15.5±3.1 | 18.5 | 34 | 27.0 | 61 | 57±9.4 |
|  |  | 2 | 16.4±3.1 | 16.6 | 33 | 28.0 |  |  |
|  |  | 3 | 13.6±3.5 | 17.4 | 31 | 30.0 |  |  |
|  |  | 4 | 8.1±2.4 | 10.9 | 19 | 42.0 |  |  |
|  |  | 5 | 5.9±1.4 | 9.1 | 15 | 46.0 |  |  |
|  |  | 6 | 8±2.8 | 14.0 | 22 | 39.0 |  |  |
|  |  | 7 | 6.2±2 | 9.8 | 16 | 45.0 |  |  |
|  |  | 8 | 8.2±2.6 | 15.8 | 24 | 37.0 |  |  |
|  |  | 9 | 10.3±2.6 | 11.7 | 22 | 39.0 |  |  |
|  |  | 10 | 6±2.7 | 12.0 | 18 | 43.0 |  |  |
| Whananaki | 2.1 | 1 | 10.2±2.6 | 14.8 | 25 | 33.6 | 58.6 | 47.9±9.9 |
|  |  | 2 | 15.6±2.4 | 12.4 | 28 | 30.6 |  |  |
|  |  | 3 | 17±2.8 | 18.0 | 35 | 23.6 |  |  |
|  |  | 4 | 9.6±2.2 | 19.4 | 29 | 29.6 |  |  |
|  |  | 5 | 12.6±2.8 | 14.4 | 27 | 31.6 |  |  |
|  |  | 6 | 10.1±1.1 | 10.9 | 21 | 37.6 |  |  |
| Whangateau | 2.3 | 1 | 10±2.4 | 12.0 | 22 | 48.8 | 70.8 | 34.6±8.1 |
|  |  | 2 | 15.1±2.6 | 13.9 | 29 | 41.8 |  |  |
|  |  | 3 | 15.2±0.9 | 16.8 | 32 | 38.8 |  |  |
|  |  | 4 | 8.9±1 | 13.1 | 22 | 48.8 |  |  |
|  |  | 5 | 15.7±2.3 | 18.3 | 34 | 36.8 |  |  |
|  |  | 6 | 9.9±2.5 | 16.1 | 26 | 44.8 |  |  |
|  |  | 7 | 10.4±1.8 | 11.6 | 22 | 48.8 |  |  |
|  |  | 8 | 8.7±1.6 | 10.3 | 19 | 51.8 |  |  |
|  |  | 9 | 8.4±2.2 | 16.6 | 25 | 45.8 |  |  |
